# Proteomic signatures of renal recovery from acute kidney injury—a translational study in critically ill postoperative patients

**DOI:** 10.1186/s40635-026-00926-0

**Published:** 2026-06-05

**Authors:** Thilo von Groote, Moritz J. Mertes, Hendrik Booke, Mahan Sadjadi, Christian Strauß, Katrin Schützenmeister, Christian Porschen, Amélie Friederike Menke, Harm-Jan de Grooth, Lui G. Forni, Melanie Meersch-Dini, Simone König, Alexander Zarbock

**Affiliations:** 1 Department of Anaesthesiology, Intensive Care and Pain Medicine, University Hospital Münster, Albert-Schweitzer-Campus 1, Building A1, 48149 Münster, Germany; 2 Department of Internal and Emergency Medicine, Nephrology and Rheumatology, University Hospital Münster, Münster, Germany; 3 Department of Intensive Care Medicine, University Medical Center Utrecht, Utrecht, The Netherlands; 4 Department of Critical Care Medicine, Royal Surrey Hospital, Guildford, UK; 5 Department of Clinical and Experimental Medicine, Faculty of Health Sciences, University of Surrey, Guildford, UK; 6 Service Unit Proteomics, University of Münster, Münster, Germany

**Keywords:** Acute kidney injury, Acute kidney disease, Proteomics, Renal recovery

## Abstract

**Background:**

Acute kidney injury (AKI) is a common complication in critically ill patients and frequently progresses to persistent renal dysfunction. However, mechanisms underlying renal recovery versus persistent dysfunction remain poorly understood. Plasma proteomic profiling may identify proteins and biological pathways associated with the development of acute kidney disease (AKD) subsequent to AKI.

**Material and methods:**

We conducted a plasma proteomics study within a prospective, single-centre observational cohort of 205 critically ill adult patients in Germany with moderate AKI (KDIGO stage 2) and predefined risk factors for AKI progression (mechanical ventilation and/or vasopressor therapy). Plasma samples from 195 patients were available for analysis in sufficient quantity. The primary outcome was AKD, defined as persistent AKI (KDIGO stage 2 or 3) or death seven days after AKI diagnosis. High-resolution mass spectrometry quantified protein abundances in non-depleted and immunodepleted plasma samples. Proteins associated with AKD were assigned using biostatistical analyses and selected candidates were validated by enzyme-linked immunosorbent assay (ELISA) in the entire cohort.

**Results:**

Out of 195 patients, 103 developed AKD (52.8%). Proteomic profiling assigned 29 proteins in undepleted plasma and 38 proteins in depleted plasma that were significantly associated with development of AKD. In undepleted plasma, gelsolin and Zinc-alpha-2-glycoprotein were prominent candidates. In depleted plasma, S100A9, a component of the Calprotectin complex, showed the strongest association with AKD development. ELISA validation demonstrated higher plasma concentrations of Calprotectin (S100A8/A9) in patients who progressed from AKI to AKD.

**Conclusions:**

Unbiased plasma proteomics revealed several proteins associated with development of AKD following moderate AKI in critically ill patients. Elevated Calprotectin (S100A8/A9) levels were independently associated with AKD development in ELISA analyses, suggesting a potential role in AKI chronification. Further studies are warranted to validate these findings and explore their mechanistic and clinical implications.

**Supplementary Information:**

The online version contains supplementary material available at 10.1186/s40635-026-00926-0.

## Introduction

Acute kidney injury (AKI) is a frequent complication in critically ill patients and is associated with increased morbidity and mortality. Patients with AKI are also at increased risk to develop chronic kidney disease (CKD) or even end-stage kidney disease (ESKD), requiring renal replacement therapy (RRT) or kidney transplantation [1, 2]. Accumulating evidence indicates that the risk of developing CKD is determined not only by AKI severity, but also by the duration of renal dysfunction [3]. This recognition has led to increased focus on time-dependent AKI trajectories, including transient versus persistent AKI and the concept of acute kidney disease (AKD), which captures ongoing renal dysfunction beyond the initial acute phase [4].

Yet, the mechanisms and pathophysiological pathways governing renal recovery versus persistent renal dysfunction after AKI remain incompletely understood, particularly in critically ill patients where AKI represents a heterogeneous clinical syndrome. Proposed mechanisms contributing to AKI persistence include tubular ischemia, epithelial cell dedifferentiation and death, impaired regenerative capacity, sustained inflammation, and progressive interstitial fibrosis [5].

In a landmark study, the RUBY trial identified urinary C–C motif chemokine ligand 14 (CCL14) as a promising biomarker of persistent AKI and this was validated in an external multicenter study [6, 7]. However, no plasma biomarker has yet been established for predicting persistent renal dysfunction after AKI, despite the practical advantages of plasma-based testing in routine intensive care.

Mass spectrometry (MS)-based proteomics enables unbiased, high-resolution analysis of the circulating proteome and has emerged as a powerful approach for mechanistic insight and potential biomarker discovery. To date, no proteomic study has specifically investigated plasma proteins associated with AKD development.

We, therefore, aimed to identify plasma proteomic signatures distinguishing patients who develop AKD from those who recover renal function within seven days following moderate AKI using a high-resolution (HR-)MS proteomic workflow for undepleted and depleted plasma. We further validated selected candidate proteins using enzyme-linked immunosorbent assays (ELISA).

## Methods

In two sets of experiments, we first used regular plasma and then repeated the experiment in plasma, which was depleted of the 14 most abundant plasma proteins, including albumin and IgGs, and investigated the protein profiles using HRMS-based comparative proteomic analyses. The goal of the first experiment was to evaluate the entire proteome in untreated plasma searching for robust disease markers. Immuno-depletion of plasma samples, on the other hand, gave access to low abundant plasma proteins; it thus provided more insight into the associated pathophysiological mechanisms of renal recovery or persistent dysfunction and potential therapeutic targets. ELISA validation was performed for the most notable proteins identified.

### Study design and study cohort

This study was performed using one set of plasma samples and clinical data of the prospective, observational single-centre “Predicting the Development of Renal Replacement Therapy Indications by Combining the Furosemide Stress Test and Chemokine (C–C Motif) Ligand 14 in a Cohort of Postsurgical Patients” study [8]. The Research Ethics Committee of the Chamber of Physicians of Westfalen-Lippe and the University of Münster approved the study prior to study commencement (2019–261-f-S). The ethics approval included an add-on study to collect plasma and urinary samples for secondary analyses. The study was conducted in accordance with the Declaration of Helsinki (Version Fortaleza, 2013). Written informed consent was obtained from all participants. If patients were unable to provide informed consent themselves due to their critical illness (e.g. due to delirium or intubation), informed consent was obtained from their legal counsels, according to local requirements and legislation.

**Fig. 1:**
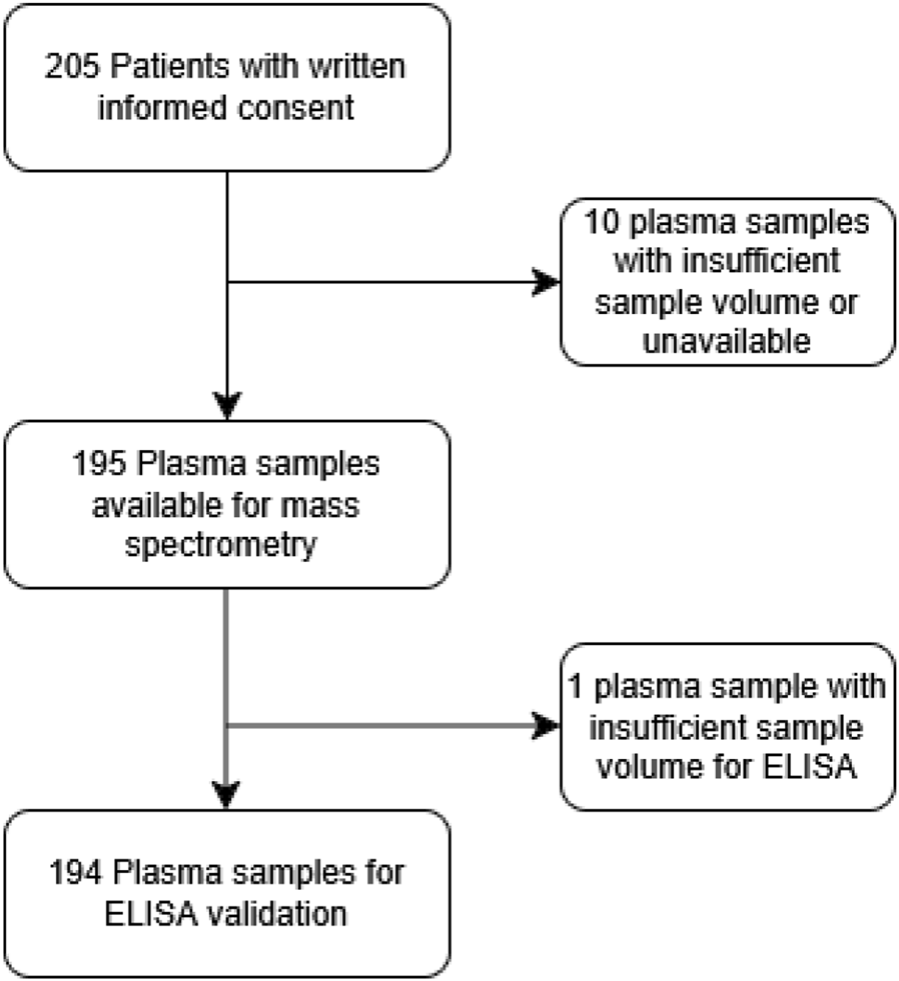
Study workflow

The study included 205 critically ill adult patients with moderate AKI (KDIGO stage 2) [9] that received a furosemide stress test (FST). Plasma baseline samples of 195 patients were available in sufficient quantities and therefore included in this study and analysed by MS. A detailed summary of the baseline characteristics is provided in Table 1. Samples were taken immediately before the furosemide stress test was conducted. In earlier studies, both mechanical ventilation and vasopressor support have been associated with higher probability of AKI as well as progression of AKI [10, 11]. Accordingly, at study inclusion, besides stage 2 AKI, patients had to be either mechanically ventilated and/or receiving vasopressors (norepinephrine/ epinephrine/ norepinephrine + epinephrine ≥ 0.1 µg/kg/min) to be eligible for inclusion. Exclusion criteria were advanced CKD with estimated glomerular filtration rate (eGFR) less than 20 mL/min/1.73 m^2^, chronic dialysis dependency, need for RRT due to drug intoxication, pregnancy or breastfeeding, or participation in an interventional trial within the last 30 days. A study workflow is provided in Fig. 1. Subsequently, one sample was excluded due to insufficient remaining volume, resulting in 194 samples from the same set being used for ELISA validation assays.

**Table 1:** Baseline patient characteristics

Characteristics	Total (*n* = 195 – Missing)	Missing, n	No AKD (*n* = 92)	AKD (*n* = 103)	*p*-value
Age, mean (SD), yr	69.82 (11.81)	0	69.25 (11.66)	70.29 (11.97)	0.540
Male Sex, n (%)	125 (64.1)	0	63 (68.50)	62 (60.2)	0.229
Weight, mean (SD), kg	83.96 (22.97)	1	86.98 (24.79)	81.30 (21.00)	0.089
BMI, mean (SD), kg/m^2^	27.73 (6.77)	13	28.26 (6.56)	27.27 (6.95)	0.325
Baseline SCr, mean (SD)	1.05 (0.46)	1	0.967 (0.393)	1.12 (0.50)	0.018
Comorbidities, *n* (%)					
Chronic kidney disease	66 (33.8)	0	25 (27.2)	41 (39.8)	0.063
Congestive heart failure	49 (25.4)	2	22 (24.2)	27 (26.5)	0.715
Arterial hypertension	137 (70.3)	0	66 (71.7)	71 (68.9)	0.669
IDDM	52 (26.7)	0	25 (27.2)	27 (26.2)	0.880
Asthma and/or COPD	40 (20.5)	0	20 (21.7)	20 (19.4)	0.689
Chronic liver disease	24 (12.4)	1	9 (9.8)	15 (14.7)	0.298
Cancer	40 (20.6)	1	21 (23.1)	19 (18.4)	0.426
Surgical specialty, *n* (%)	191 (100)	4			0.099
Cardiothoracic	97 (50.8)		42 (47.2)	55 (53.9)	
General	32 (16.8)		18 (20.2)	14 (13.7)	
Neurosurgery	17 (8.9)		12 (13.5)	5 (4.9)	
Trauma	14 (7.3)		8 (9)	6 (5.9)	
Gynaecology or Urology	9 (4.7)		2 (2.2)	7 (6.9)	
Orthopaedics	1 (0.5)		0 (0)	1 (0.5)	
Other	21 (11.0)		7 (7.9)	14 (13.7)	
Surgical characteristics, *n* (%)					
Emergency surgery	115 (59.0)	2	54 (59.3)	61 (59.8)	0.948
Transplantation surgery	7 (3.6)	0	2 (2.2)	5 (4.9)	0.315
ICU Characteristics					
APACHE Score, median (Q1, Q3)	27 (22, 35)	0	25.5 (20.5, 31.5)	31 (24, 36)	< 0.001
SOFA Score, median (Q1, Q3)	12 (9, 14)	0	10 (8, 12)	13 (10, 16)	< 0.001
Mechanical ventilation*, *n* (%)	165 (84.6)	0	73 (79.3)	92 (89.3)	0.054
Vasopressors*, *n* (%)	187 (95.9)	0	86 (93.5)	101 (98.1)	0.107
SIRS/Sepsis*, *n* (%)	99 (50.8)	0	45 (48.9)	54 (52.4)	0.624
Blood transfusion**, *n* (%)	44 (22.6)	0	17 (18.5)	27 (26.2)	0.197

^*^ During sample collection; ** Within 6 h before or during sample collection; *APACHE* Acute Physiology And Chronic Health Evaluation, *BMI* Body Mass Index, *COPD* chronic obstructive pulmonary disease, *ICU* Intensive Care Unit, *IDDM* Insulin Dependent Diabetes Mellitus, *SCr* Serum Creatinine, *SIRS* Systemic Inflammatory Response Syndrome, *SOFA* Sequential Organ Failure Assessment

### Data collection and definitions

Data were collected using the study electronic case report file (eCRF) and analysed using IBM SPSS Statistics 29.0.0.0(241). The primary outcome was acute kidney disease (AKD), defined as either persistence of moderate or severe AKI according to the KDIGO criteria (stage 2 or 3) at day 7 after study inclusion (KDIGO stage 2), using both urinary and serum creatinine criteria, or death.

### Protein isolation and MS-based analysis

Plasma samples (5 µl) were processed according to an established protocol for filter-aided tryptic digestion including reduction and alkylation[12]; they were finally dissolved in 10 µl 0.1% formic acid containing 5% acetonitrile for reversed-phase liquid chromatography (LC) coupled to high definition (HD)MS. In a second set of experiments, samples (10 µl) were depleted using Top14 High Select mini depletion spin columns (ThermoFisher Scientific) according to the instructions of the manufacturer. They were subsequently also digested in the same manner as the first set. Peptide solutions (3 and 1 µl, respectively) were analysed by LC-HDMS with Synapt G2 Si / M-Class nanoUPLC (Waters Corp., Manchester, UK) using C18 µPAC columns (trapping and 50 cm analytical; PharmaFluidics, Ghent, Belgium) with a 90 min gradient (solvent system 100% water versus 100% acetonitrile, both containing 0.1% formic acid).

### Data analysis and statistical methods

Data were analysed with Progenesis for Proteomics (QIP4.2.7207.22925, Nonlinear Diagnostics/Waters Corp.) using the human Uniprot database (UP000005640) with false discovery rate correction [12]. Shortlists of the protein output were created by demanding protein assignment by at least two peptides, a fold value of at least 2 and a significance of *p*-value ≤ 0.05. Network and gene ontology (GO) analyses were performed using the String database (String Consortium 2021, ELIXIR Core Data Resource) and Enrichr software (Center for Bioinformatics, Icahn School of Medicine at Mount Sinai). Statistical analyses were performed using IBM SPSS Statistics (Version 31, IBM Corp., Armonk, NY, USA). The distribution of Calprotectin (S100A8/A9) was assessed using the Shapiro–Wilk test. Due to non-normal data-distribution (*p* < 0.001), group comparisons were conducted using the Mann–Whitney U test. A univariate regression analysis was performed to evaluate the association between levels of Calprotectin (S100A8/A9) and developing AKD. Calprotectin (S100A8/A9) values were log-transformed to reduce data skewness. To control for potential confounding by systemic inflammation, a multivariate logistic regression was performed including SIRS/Sepsis at the time of sample collection as a covariate. Area under the Receiver operating characteristic (ROC) curve of Calprotectin (S100A8/A9) for predicting AKI-to-AKD progression was performed. Finally, the optimal cutoff for Calprotectin (S100A8/A9) was determined using the Youden-index.

### Validation of proteins using ELISA

ELISA analyses were performed on the same patient cohort as MS analyses; yet, one patient did not have enough sample volume for consecutive ELISA analysis (MS: *n* = 195; ELISA: *n* = 194). Blood samples were collected by standard methods on the day of enrolment prior to FST. The samples were centrifuged and supernatants were stored at − 80°C until assayed. All mediators (S100A8/A9, Zinc-alpha-2-glycoprotein and Gelsolin) were analysed using commercial available ELISA kits (Human S100A8/ S100A9 ELISA Kit, Abcam, #ab267628; Human Zinc-alpha-2-glycoprotein ELISA Kit, Abcam, #ab277419 and Human Gelsolin ELISA Kit, Abcam, #ab270215). The S100A8/A9 ELISA specifically quantifies the heterodimer (Calprotectin), which represents the predominant biologically active circulating form, whereas the MS data reflects total S100A9 abundance independent of complex formation.

## Results

### Patient cohort and baseline characteristics

A total of 195 critically ill postoperative patients with KDIGO stage 2 AKI and mechanical ventilation and/or vasopressors were included in the study. Of these, 103 patients progressed to AKD and/or died, while 92 patients recovered within 7 days (no AKD). Baseline clinical characteristics are summarized in Table 1. A study flowchart summarizing patient selection, exclusions, and sample allocation is provided in Fig. 1. MS-based discovery proteomics was performed on 195 plasma samples, and ELISA validation was performed on 194 samples (same cohort, except one sample unavailable for ELISA). Comparative proteomics analyses were carried out in undepleted and depleted plasma samples of patients with AKD or death and without AKD or death (noAKD) following AKI. Table 2 displays the shortlisted protein hits (assignment by at least two peptides, fold value > 2, FDR-adjusted p-value ≤ 0.05).

**Table 2:** Significantly changed protein matches in undepleted and depleted plasma described with at least two unique peptides. For data, see the Supplementary Excel File

Accession	Name	Description	Peptide count	Unique peptides	Max fold change
Undepleted					
P25311	AZGP1	Zinc-alpha-2-glycoprotein	36	31	2.20
P00736	C1R	Complement C1r subcomponent	30	26	2.33
P69905	HBA1	Hemoglobin subunit alpha	30	24	2.02
P06396	GSN	Gelsolin	21	16	4.01
Q7Z3Y8	KRT27	Keratin type I cytoskeletal 27	10	5	4.00
A0A0C4DH41	IGHV4-61	Immunoglobulin heavy variable 4–61	9	2	6.95
P02144	MB	Myoglobin	8	8	2.84
Q8IYD8	FANCM	Fanconi anemia group M protein	8	5	4.59
P08133	ANXA6	Annexin A6	6	2	3.70
Q9UPN9	TRIM33	E3 ubiquitin-protein ligase TRIM33	5	4	3.98
Q86VB7	CD163	Scavenger receptor cysteine-rich type 1 protein M130	5	4	7.65
Q86W28	NLRP8	NACHT LRR and PYD domains-containing protein 8	5	2	2.12
A0A075B6I0	IGLV8-61	Immunoglobulin lambda variable 8–61	3	3	3.44
P27816	MAP4	Microtubule-associated protein 4	4	2	9.27
Q9NSI8	SAMSN1	SAM domain-containing protein SAMSN-1	3	2	2.31
Q9H3Q1	CDC42EP4	Cdc42 effector protein 4	2	2	11.69
P78412	IRX6	Iroquois-class homeodomain protein IRX-6	4	3	3.68
Q9H2U2	PPA2	Inorganic pyrophosphatase 2 (mitochondrial)	3	2	2.18
P01701	IGLV1-51	Immunoglobulin lambda variable 1–51	3	3	9.33
Q9NQ30	ESM1	Endothelial cell-specific molecule 1	2	2	5.84
O95445	APOM	Apolipoprotein M	2	2	6.25
Depleted					
P68871	HBB	Hemoglobin subunit beta	55	39	2.31
P23141	CES1	Liver carboxylesterase 1	34	26	3.38
P09467	FBP1	Fructose-1–6-bisphosphatase 1	19	14	2.98
P02771	AFP	Alpha-fetoprotein	18	13	3.12
P06702	S100A9	Protein S100-A9	11	8	2.48
P02144	MB	Myoglobin	11	8	10.11
P63104	YWHAZ	14–3-3 protein zeta/delta	12	6	4.78
P61981	YWHAG	14–3-3 protein gamma	6	3	2.06
P50395	GDI2	Rab GDP dissociation inhibitor beta	9	6	3.14
P0CF74	IGLC6	Immunoglobulin lambda constant 6	7	2	80.78
Q13228	SELENBP1	Methanethiol oxidase	10	6	2.30
P00441	SOD1	Superoxide dismutase [Cu–Zn]	10	7	3.57
O43866	CD5L	CD5 antigen-like	7	5	2.49
P41222	PTGDS	Prostaglandin-H2 D-isomerase	8	5	6.02
P80188	LCN2	Neutrophil gelatinase-associated lipocalin	7	5	2.30
P00966	ASS1	Argininosuccinate synthase	5	3	8.01
Q15323	KRT31	Keratin_ type I cuticular Ha1	7	3	5.57
P14550	AKR1A1	Aldo–keto reductase family 1 member A1	7	5	2.16
P05089	ARG1	Arginase-1	4	4	4.86
P48681	NES	Nestin	4	2	5.78
P50440	GATM	Glycine amidinotransferase_ mitochondrial	5	2	274.40
Q9BTM1	H2AJ	Histone H2A.J	3	2	14.02
P0DME0	SETSIP	Protein SETSIP	3	2	2.23
P10720	PF4V1	Platelet factor 4 variant	5	3	2.73
Q93099	HGD	Homogentisate 1_2-dioxygenase	3	3	4.29
Q9NSE4	IARS2	Isoleucine—tRNA ligase (mitochondrial)	4	2	5.69
Q96PC3	AP1S3	AP-1 complex subunit sigma-3	3	2	2.93
P17516	AKR1C4	Aldo–keto reductase family 1 member C4	3	3	12.83
Q12918	KLRB1	Killer cell lectin-like receptor subfamily B member 1	2	2	2.11

### Mass spectrometry discovery and PCA analysis

HR-MS based discovery identified 62 proteins with fold-change ≥ 2 and FDR-adjusted *p*-value < 0.05 between AKD and no AKD patients. Principal component analysis (PCA) revealed partial separation between AKD and no-AKD groups, with some overlap reflecting patient heterogeneity. PCA results are provided in Supplementary Fig. 1.

### Undepleted plasma

**Fig. 2:**
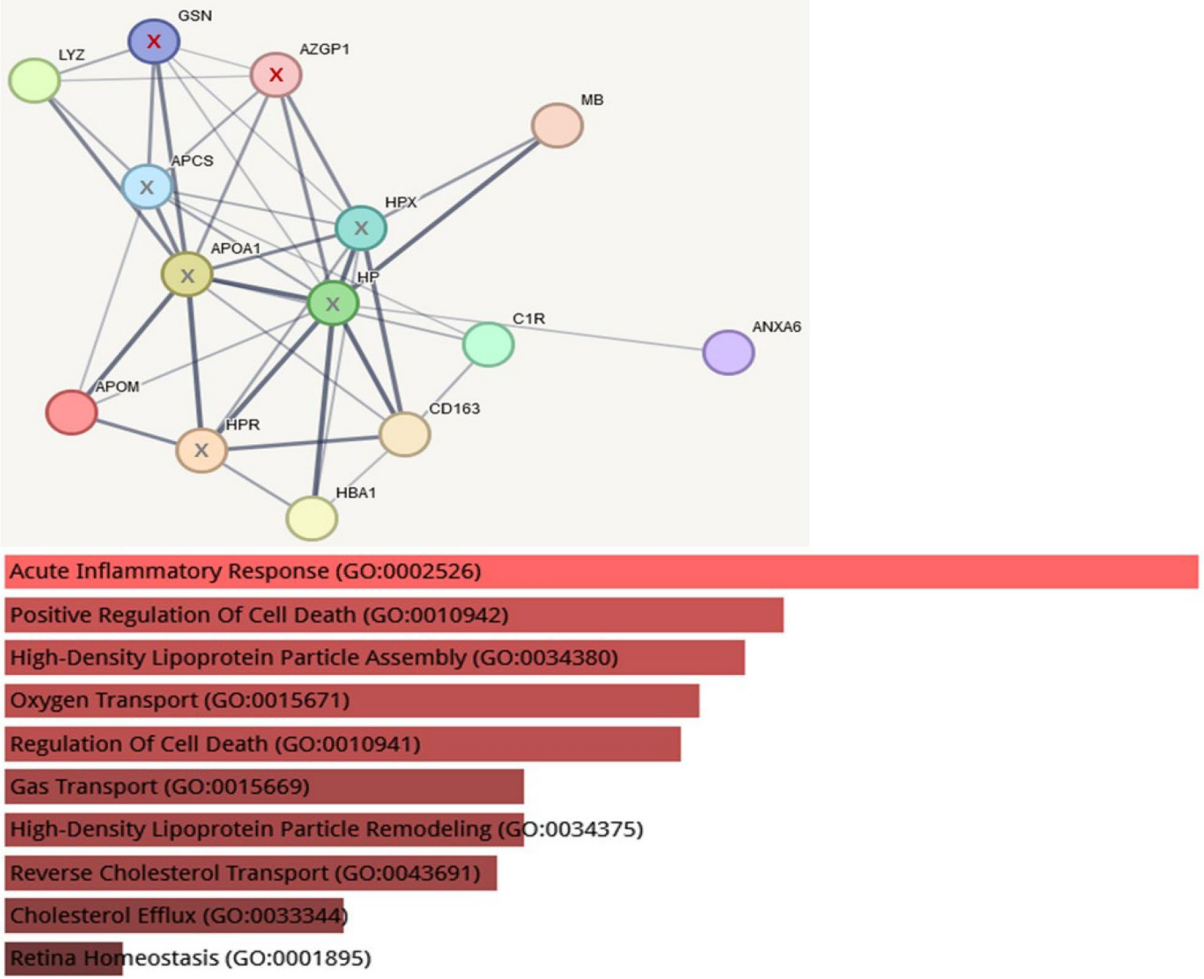
String network and Enrichr GO (GO terms for subanalysis GO Biological Process 2023) analyses of proteins detected with significantly more abundance in AKD than in noAKD undepleted samples. Proteins of special interest (GSN, AZGP1) are marked with a red x. The other depicted members of the network (*HP* haptoglobin, *HPR* haptoglobin-related protein, serum amyloid P-component, *APCS* apolipoprotein A-I, *APOA1* hemopexin, *HPX* marked with x) were not significantly changed in the experiment

The comparison of the detected proteins in the experiments with undepleted plasma revealed significant changes in acute-phase, inflammatory and defense response proteins. The String network illustrating the involved proteins and the results for gene ontology analysis are shown in Fig. 2. Gelsolin (GSN) was measured with fourfold more abundance in AKD than in noAKD samples, the fold values for the other depicted proteins (Zinc-α2-glycoprotein, AZGP1; hemoglobin α, HBA1; complement C1r, C1R; myoglobin, MB; annexin A6; ANX6; scavenger receptor cysteine-rich type 1 protein M130, CD163; apolipoprotein M, APOM; lysozyme C, LYZ) were slightly lower.

### Depleted plasma

**Fig. 3:**
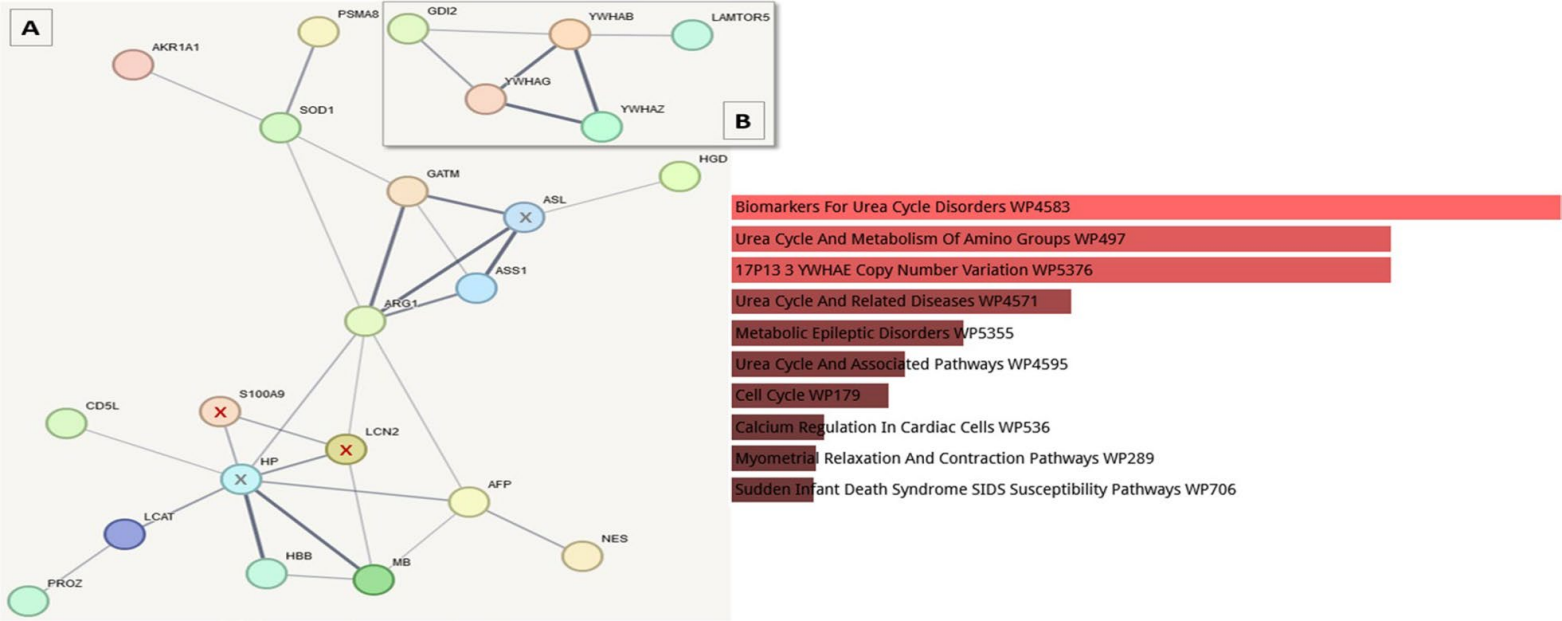
String network and Enrichr GO (subanalysis WikiPathway 2023 Human) analyses of proteins detected with significantly more abundance in AKD than in noAKD depleted samples. The proteins of special interest (S100A9, LCN2) are marked with a red x. The other depicted members of network A (HP; argininosuccinate lyase, ASL; marked with x) were not significantly changed in the experiment. Network A: hemoglobin β, HBB; α-fetoprotein, AFP; S100A9; superoxide dismutase, SOD1; CD5 antigen-like, CD5L; LCN2; arginosuccinate synthase, ASS1; aldo–keto reductase family 1 member A1, AKR1A1; arginase-1, ARG1; proteasome subunit α-type 8, PSMA8; nestin, NES; homogentisate 1,2-dioxygenase, HGD; vitamin K-dependent protein Z, PROZ; Phosphatidylcholine-sterol acyltransferase, LCAT; MB. Network B: 14–3-3 proteins α/β, γ, ζ/δ, YWHAB, YWHAG, YWHAZ; Rab GDP dissociation inhibitor β, GDI2; regulator complex protein LAMTOR5, LAMTOR5

Significant changes were observed for proteins involved in urea cycle disorders and acute kidney tubular necrosis. Proteins of interest include the well-established marker protein neutrophil gelatinase-associated lipocalin (LCN2, also known as NGAL) and protein S100A9 (S100A9, part of the Calprotectin (S100A8/A9) protein complex). The second member of the Calprotectin complex, S100A8, was not significantly changed in our proteomic experiments. Figure 3a visualises results of the depleted protein analyses. A separate network was formed by 14–3-3 proteins (Fig. 3b).

### ELISA Validation of Calprotectin (S100A8/A9)

**Fig. 4:**
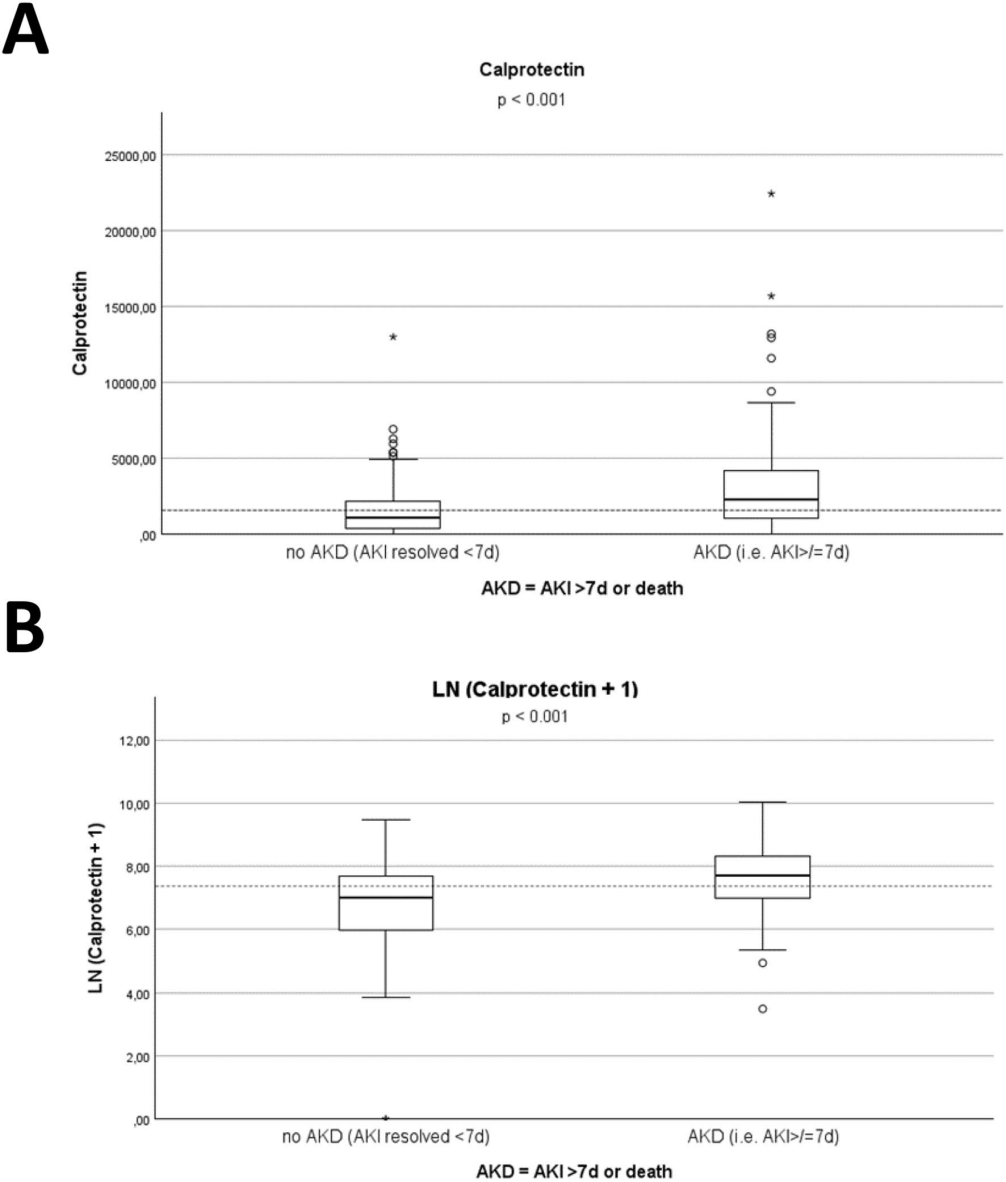
Boxplots of Calprotectin-(S100A8/A9) concentrations (ng/ml), data presented as mean (stripped line), mean per group (fat lines), interquartile range (IQR), range. **A** raw values; **B** log-transformed values [Ln(Calprotectin + 1)]

**Fig. 5:**
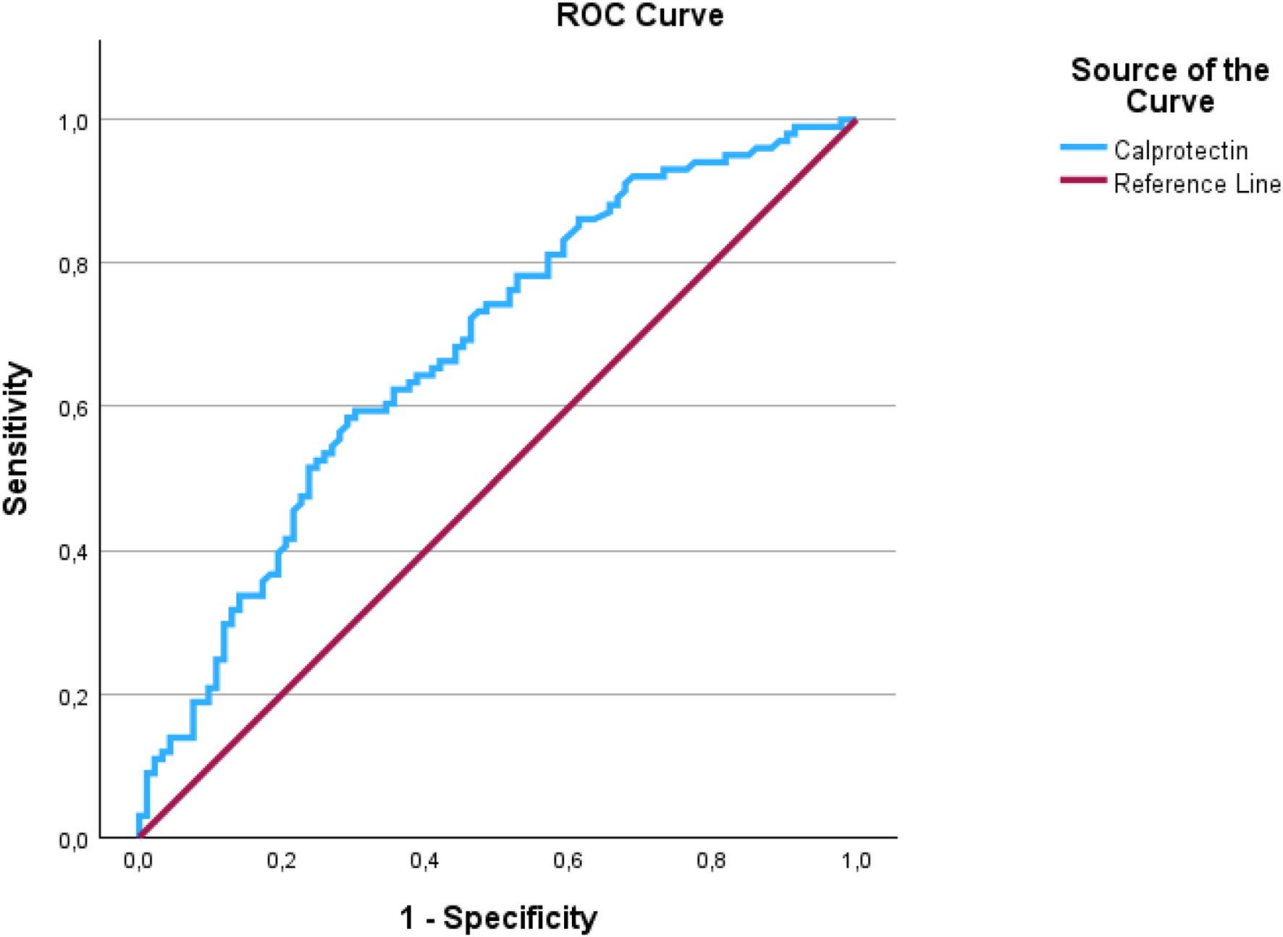
Receiver operating characteristic (ROC) curve of Calprotectin-(S100A8/A9) for predicting AKI-to-AKD progression, with an area under the curve (AUC) of 0.680 (95% CI 0.605–0.755), *p* < 0.001. The optimal cut-off for Calprotectin-(S100A8/A9) was determined using the Youden index (J = Sensitivity + Specificity − 1). The maximal Youden index was 0.294 at a threshold of 1997,51 ng/ml, yielding a sensitivity of 58% and specificity of 71%

ELISA showed equal distributions of gelsolin and Zinc-alpha-2 among patients with and without progression to AKD. However, patients who developed AKD had significantly higher levels of Calprotectin (S100A8/A9) in ELISA measurements compared to those without AKD (median [IQR]: 2248 ng/ml [1065—4180] vs 1094 ng/ml [391 – 2221]), *p* < 0.001 (Mann–Whitney-U test). In the univariate logistic regression using untransformed Calprotectin (S100A8/A9) concentrations, the effect estimate approached zero, resulting in an odds ratio of 1.00 (95% CI 1.00–1.00), reflecting the absence of interpretable change per one-unit increase given the wide range of measured values. Accordingly, analysis with log-transformed Calprotectin (S100A8/A9) values revealed a statistically significant association with progression to AKD. Higher ln-Calprotectin (S100A8/A9) levels were associated with increased odds of developing AKD, with an OR of 1.78 (95% CI 1.35–2.35), p < 0.001. Each unit increase in ln-Calprotectin (S100A8/A9) corresponding to an approximate 2.7-fold rise in the biomarker, was associated with a 78% increase in the odds of AKD development (Fig. 4). After adjusting for the presence of SIRS/sepsis at the time of sampling, log-transformed Calprotectin (S100A8/A9) remained a predictor of AKD (adjusted OR 1.8, 95% CI 1.36 – 2.4, *p* < 0.001), indicating that this association is independent of systemic inflammation. The ROC analysis demonstrated a low-moderate discriminative ability of Calprotectin (S100A8/A9) for identifying patients who progressed from AKI to AKD, with an AUC of 0.680 (95% CI 0.605–0.755). The optimal cut-off determined by the maximal Youden index (0.294) was 1997.5 ng/mL, yielding a sensitivity of 58% and a specificity of 71% (Fig. 5).

## Discussion

In this comprehensive plasma proteomics study of critically ill patients with moderate AKI, we assigned several proteins associated with persistent renal dysfunction seven days after diagnosis (AKD). Using unbiased HRMS in both undepleted and immunodepleted plasma, we detected protein signatures linked to the AKI-to-AKD transition. Proteins with increased expression in patients with AKD included GSN, AZGP1 and S100A9. Among these, S100A9 (part of the Calprotectin-complex) emerged as the most consistently associated protein and remained independently associated with AKD after adjustment for systemic inflammation, suggesting a potential role in AKI chronification.

AKI is a highly heterogeneous clinical syndrome, particularly in critically ill patients, and this heterogeneity was reflected in our proteomic data. Principal component analyses demonstrated only partial separation between patients with and without AKD, which is not unexpected given the diversity of underlying diseases, comorbidities, and causes of AKI in this cohort. Importantly, this heterogeneity mirrors real-world intensive care populations and supports the generalisability of our findings. Rather than identifying a single dominant biomarker, our results highlight biological pathways that may contribute to persistent renal dysfunction following AKI.

In undepleted plasma, we assigned 29 proteins that were associated with AKD. We detected two important hits, which have been described in the context of renal disease before, namely gelsolin (GSN) and Zinc-alpha-2-glycoprotein (AZGP1). GSN, a multifunctional actin-modulating protein is a key component of the extracellular scavenger system and influences cellular motility [13]. GSN was linked to podocytopathy in CKD before and may influence immune response to inflammation in CKD and other diseases (such as acute liver failure, myocardial infarction, or septic shock) by unknown mechanisms [14, 15]. Our findings are in line with reports of a prospective, observational study in patients undergoing cardiac catheterization that found significantly elevated levels of plasma GSN in patients who developed AKD [16]. This suggests both a potential direct role in nephropathy of the AKI-to-CKD continuum via AKD, as well as a potential role in critical illness overall which requires further investigation.

AZGP1, a freely filtered glycoprotein cleared by tubular uptake, accumulates during reductions in glomerular filtration and has been linked to fibrosis modulation through effects on epithelial differentiation and TGF-β signalling, which represents a major pathway of persistent AKI[17]. Significantly elevated levels of AZGP1 have been described in patients with stage 3 AKI and in chronic haemodialysis patients [18]. Experimental studies suggest antifibrotic and nephroprotective properties of AZGP1, indicating a complex and context-dependent role in kidney injury and repair.^15^ In a recent study, treatment with AZGP1 was investigated in mice with unilateral ureteric obstruction (as a model of post-renal AKI) and found better preservation of tubular integrity, reduced collagen deposition and lower expression of injury and fibrosis markers in mice treated with AZGP1 at 14 days after urethral obstruction [19].

Despite their assignment in the proteomic discovery phase, neither gelsolin nor AZGP1 discriminated patients who progressed to AKD when measured by ELISA in the full cohort. These findings contrast with Kuo et al., who reported that early post-procedure urinary gelsolin ratios were independently associated with subsequent AKD development following cardiac catheterization [16]. Similarly, Zinc-alpha-2-glycoprotein has been shown to be elevated during AKI and to correlate with extra-renal complications in the acute phase, and higher circulating levels have been linked to chronic kidney disease progression in diabetic populations [20]. Differences in patient populations, timing of sampling, disease severity, and outcome definitions may explain these discrepancies. Importantly, these results underscore that proteins involved in AKI pathophysiology are not necessarily suitable as standalone biomarkers for AKD risk stratification.

In depleted plasma, we assigned a distinct set of 38 proteins associated with AKD. Interestingly, S100A9, a protein of the Calprotectin complex (S100A8/S100A9) was more strongly expressed in patients who developed AKD. The calcium-binding proteins S100A8/A9 are primarily expressed in neutrophils and monocytes and can be released into the extracellular space as damage associated molecular patterns (DAMPs) in response inflammatory stimuli. They form both homodimers and heterodimers, with the latter (Calprotectin) mediating immune-regulatory functions [21]. Previous studies identified a potential role of the S100A8/A9 complex in AKI: For example, using single-cell RNA sequencing. Yao et al. described kidney infiltration with of S100A8/A9^+^ macrophages in AKI and the relevance of renal S100A8/A9 to tissue injury [22]. In a bilateral Ischemia–Reperfusion-Injury model of AKI, targeting the S100A8/A9 signalling with small-molecule inhibitors exhibited nephroprotective effects represented by improved renal function and reduced mortality, and decreased inflammatory response, ameliorated kidney injury. Of interest for our study findings, this study also reported improved long-term outcomes with decreased renal fibrosis. The findings of Yao et al. support S100A8/A9 blockade as a potential target for patients with AKI to reduce kidney injury and fibrosis. A recent study in mice with septic AKI investigated the effect of a S100A9 inhibitor that prevents S100A8/A9 to bind to Toll-like receptor 4 [23]. This study found significantly reduced renal-dysfunction and pathological alterations in mice treated with the S100A9 inhibitor. The authors detected evidence that this effect was due to reduced sepsis-associated AKI induced renal tubular epithelial cell apoptosis, inflammation, superoxide production and mitochondrial dynamic imbalance. Clinical studies also investigated plasma S100A8/A9 complex in the context of AKI. In an observational study, Nikolakopoulou et al. reported a significant increase in S100A8/A9 after cardiopulmonary bypass surgery and S100A8/A9 levels were significantly higher in patients who developed AKI postoperatively (AUC 0.81 (95% CI: 0.676–0.949)) [24]. Why only S100A9, but not S100A8, was significantly associated with AKD in our samples is unclear, however, a previous study focussing on sepsis-associated AKI also described a linkage to S100A9, but not S100A8 [25]. We subsequently used ELISA to validate Calprotectin in our cohort. Elevated plasma Calprotectin levels were independently associated with AKD, even after adjustment for SIRS or sepsis, indicating that this association might not merely be a reflection of systemic inflammation. These findings should nevertheless be interpreted with caution, as baseline differences in illness severity and renal function between groups may have contributed to the observed associations (Table 1).

Despite this biologic relevance, the discriminative performance of Calprotectin for predicting AKI-to-AKD progression was modest. This finding highlights that the molecular mechanisms driving persistence of renal dysfunction likely differ from those underlying the initial development of AKI and may not be adequately captured by one-time, single circulating biomarkers. Instead, AKD may represent the cumulative result of sustained inflammation, metabolic dysregulation, impaired tissue repair and induced fibrosis, which are most likely reflected by the presence of various mediators rather than individual proteins.

While we focussed on S100A9 and Calprotectin respectively, many other of the shortlisted proteins have been discussed in the context of renal disease, e.g., superoxide dismutase [26], protein Z [27], Complement C1r serine protease [28], Apolipoprotein M [29] and lysozyme [30, 31]. Finally, in the depleted plasma samples, 14–3-3 proteins formed a separate network. These are known to be upregulated in renal pathologies [32, 33]. Other assigned proteins expressed lower fold-values or were considered probably not relevant to the development of AKD, based on literature review.

Major strengths of this study include the unbiased proteomic approach, the large sample size, and the inclusion of a heterogeneous cohort representative of critically ill (surgical) patients with AKI. By analysing both undepleted and depleted plasma, we were able to capture changes in abundant circulating proteins as well as lower-abundance proteins potentially involved in disease mechanisms. Importantly, candidate proteins were validated using orthogonal methods in the entire cohort.

Our study also has several limitations that must be acknowledged. First, although adjustment for SIRS/sepsis was performed, the observed association between Calprotectin (S100A8/A9) and progression to AKD or death should be interpreted with caution. Baseline differences in illness severity and renal function between groups (Table 1) suggest the possibility of residual confounding that may not be fully captured by the adjusted analysis. Accordingly, we cannot exclude the influence of broader illness severity, preoperative renal impairment, and other unmeasured perioperative factors on the observed associations. These findings therefore support an association rather than definitive independence of Calprotectin from other determinants of postoperative AKI risk. Second, the data is derived from a single-centre study, thereby potentially limiting generalisability of results. Third, our study focuses on a highly selected postoperative ICU population with KDIGO stage 2 AKI who require mechanical ventilation and/or vasopressors and underwent a furosemide stress test. Within this cohort, half of the patients had undergone cardiac surgery. Furthermore, patients had high severity of disease as indicated by the median APACHE and SOFA scores. This selection defines a population with severe illness and hemodynamic instability, likely enriching for ischemic, cardiac-surgery, or sepsis-associated mechanisms of kidney injury. Other common aetiologies, such as nephrotoxic injury in the absence of significant hemodynamic compromise are likely underrepresented. Consequently, the results should be interpreted in the context of this specific, high-risk ICU phenotype, and caution is warranted when extrapolating to the broader ICU population. Third, our study is exploratory, and multiple testing and high volume of data analysed increases risk for false-positive findings, although stringent identification criteria and cut-offs were used and results validated by ELISA. When interpreting our results, it must be considered that no healthy control group was used, but that we compared critically ill patients with a favourable renal outcome with critically ill patients with an unfavourable renal outcome. Hence, other factors of systemic illness, inflammation and other prognostic factors may be present in the study cohort, but this is representative of critically ill patients with AKI. A further limitation concerns the detection of hemoglobin subunit alpha (HBA1) among the differentially abundant proteins in AKD patients. Hemoglobin-derived proteins in serum are commonly considered markers of hemolysis, which may occur in vivo, during blood collection, handling, or sample processing. Hence, the finding of elevated HBA1 should be interpreted with caution, and future prospective studies should incorporate systematic hemolysis monitoring. Similarly, keratin was among the top hits in our study, which could also potentially represent a contamination. While these technical factors cannot be fully excluded, they are unlikely to affect the primary conclusions regarding AKD-associated proteins.

Importantly, the proteomic analysis quantified individual abundance of S100A8 and S100A9 proteins respectively, whereas the ELISA specifically measured calprotectin (the S100A8/A9 heterodimer), as this is the predominant and biologically active circulating form of S100A9 and represents the most clinically established analyte [34]. The observed differences between MS-based discovery and ELISA validation for Gelsolin and Zinc-alpha-2-glycoprotein likely reflect both methodological and biological factors: MS quantifies enzymatically generated peptides rather than intact proteins, and signal intensity depends on peptide detectability, ionization efficiency, and sequence coverage. MS also does not differentiate isoforms, proteolytic fragments, or higher-order complexes. In contrast, ELISA measures total protein concentrations using antibodies directed against specific epitopes of the intact protein (or heterodimer for S100A8/A9). Differences in dynamic range or pre-analytical variability (e.g., storage, freeze–thaw cycles) may further contribute to discrepancies. Future studies should consider quantifying both the individual subunits and the S100A8/A9 complex in parallel to better understand their respective biological and clinical contributions. Furthermore, MS-based proteomic analyses have inherent limitations with regard to the detection of low abundant proteins and the measurement of proteoforms^37^. Appropriate methods mitigated this issue, for example using algorithm-based quality criteria (confidence of assignment, false-positive rate), sensible cut-offs of least number of peptides matched and manual data inspection. Such total proteome analyses require subsequent data validation which we have performed on three selected proteins of interest. Our findings offer insights into potential pathophysiological mechanisms of AKI chronification (AKI-to-AKD transition), but do not demonstrate sufficient predictive performance for accurate prediction of AKD as a stand-alone biomarker.

## Conclusion

In conclusion, this comprehensive plasma proteomics study assigned multiple proteins associated with persistent renal dysfunction following moderate AKI in critically ill patients. Elevated plasma Calprotectin (S100A8/A9) was independently associated with the development of AKD, highlighting a role for sustained innate immune activation in the AKI-to-AKD transition. In addition, gelsolin and Zinc-alpha-2-glycoprotein were highlighted in the unbiased proteomic discovery phase as proteins linked to AKD and have previously been implicated in renal injury, immune modulation, and fibrotic pathways. Although neither gelsolin, nor Zinc-alpha-2-glycoprotein discriminated AKD development in ELISA-based validation, their detection underscores the complex and multifactorial biology underlying renal recovery versus injury persistence after AKI. Together, these findings advance the understanding of AKI chronification, highlight inflammatory and repair-related pathways involved in AKD, and provide a rationale for future mechanistic studies and multimarker strategies aimed at improving risk stratification and therapeutic targeting in patients with AKI.

## Supplementary Information

Supplementary Material 1.

## Data Availability

All data are provided upon reasonable request.
