# Supplementary figures and images for: Proteomic signatures of renal recovery from acute kidney injury—a translational study in critically ill postoperative patients
Source: Intensive Care Med Exp. 2026 Jun 5;14:68. doi: 10.1186/s40635-026-00926-0 (PMC13241338; doi:10.1186/s40635-026-00926-0)

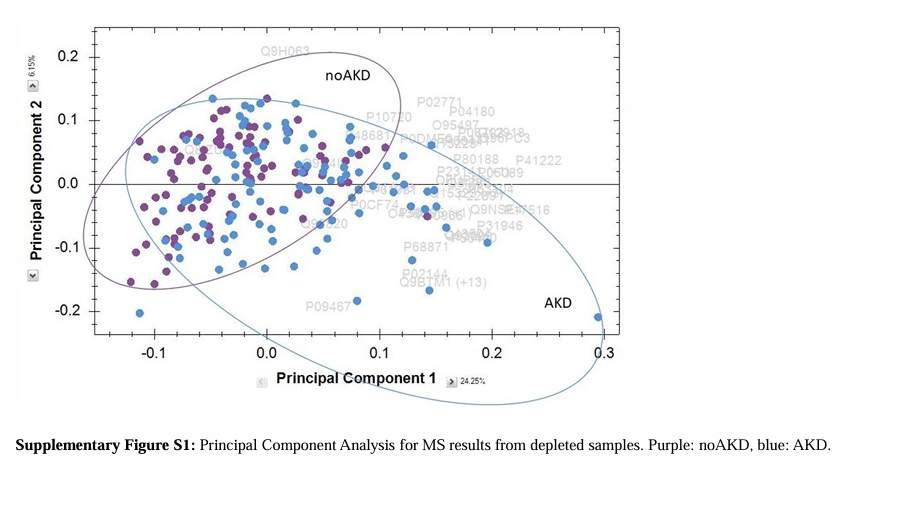

Supplement: Supplementary file 1 — Supplementary Material 1. [file 40635_2026_926_MOESM1_ESM.docx]
